# Does the addition of AMACR to CK20 help to diagnose challenging cases of urothelial carcinoma in situ?

**DOI:** 10.1186/s13000-019-0871-8

**Published:** 2019-08-16

**Authors:** Erin L. J. Alston, Debra L. Zynger

**Affiliations:** Department of Pathology, The Ohio State University Medical Center, 410 W 10th Ave., 401 Doan Hall, Columbus, OH 43210 USA

**Keywords:** Carcinoma in situ, Bladder, Immunohistochemistry, AMACR, CK20

## Abstract

**Background:**

Urothelial carcinoma in situ (CIS) in the bladder can be difficult to diagnose due to factors including procedural artifact, minimal tissue sampled, therapy-related changes, and various CIS growth patterns. Prior data has demonstrated an increase in alpha-methylacyl-CoA-racemase (AMACR) in urothelial CIS, but there is no information on its utility for diagnosing difficult cases. The aim of this investigation was to assess the expression of AMACR that was ordered on equivocal bladder cases during clinical practice.

**Methods:**

Transurethral resections of the bladder in which AMACR and CK20 were performed during diagnostic workup were identified and cases with a final diagnosis of CIS (*n* = 22) or non-neoplastic urothelium (*n* = 30) were selected. Additionally, cases in which a diagnosis of CIS was rendered without IHC (*n* = 20) were selected and tested for AMACR expression.

**Results:**

Sensitivity of AMACR for CIS diagnosed with IHC during clinical practice was 73% and specificity was 97%, while CK20 was 95% sensitive and 80% specific. Sensitivity of AMACR in CIS diagnosed without IHC was 100%. In all groups, AMACR had inconsistent intensity, compared to CK20 which had consistent, strong intensity.

**Conclusions:**

AMACR was usually positive in urothelial CIS and negative in non-neoplastic urothelium. However, it is important to note that AMACR was less sensitive in difficult cases, while CK20 was more sensitive with more consistent, strong staining compared to AMACR.

## Background

Urothelial carcinoma in situ (CIS) is a high-risk subtype of non-invasive bladder cancer that has a substantial rate of invasion and death, with a 5-year risk of progression up to 45% [1–3]. However, if appropriate intervention is made, risk of progression is significantly reduced with improved disease-free survival [3]. It is important to accurately identify this lesion in a timely manner so that appropriate treatment can be administered. The initial treatment is typically intravesical Bacillus Calmette-Guerin (BCG) therapy. For refractory urothelial CIS, radical cystectomy is the recommended approach [4–6]. Thus, it is essential to distinguish between CIS and non-neoplastic tissue because underdiagnosis can lead to invasion, while overdiagnosis can lead to harmful and unnecessary treatment.

Distinguishing urothelial CIS from non-neoplastic urothelium using hematoxylin and eosin (H&E) staining alone can be difficult for multiple reasons including denudation of tissue, artifact from procedure (fragmentation, crush, and/or cautery), inadequate sample size, or therapy-related changes. Furthermore, some morphologic growth patterns of urothelial CIS can be difficult to diagnose, such as pagetoid spread or clinging carcinoma [3]. Utilization of immunohistochemistry (IHC) can be helpful in difficult-to-diagnose cases of CIS. Immunostains that have been shown to be of some benefit in diagnosis include CK20 [7–14], CD44s [7, 13], Ki67 [7, 10–12], and p53 [7, 8, 12–14]. Markers such as CD44s, Ki67, and p53 are problematic due to overlapping expression profiles of CIS and reactive nonneoplastic urothelium.

AMACR has been found to be overexpressed in urothelial carcinoma, with amount of expression correlating with tumor grade [15–18]. Like CK20, prior studies described increased expression of AMACR in urothelial CIS [19, 20]. Based on this these publications, our institution has utilized AMACR in addition to CK20 in challenging cases. However, there are currently no studies that evaluate the expression of AMACR in cases that were equivocal on H&E in which staining was performed during clinical practice. The aim of this investigation was to assess the expression of AMACR in difficult bladder cases in which testing was performed in clinical practice and compare its utility to that of CK20 for diagnostic urothelial CIS.

## Methods

### Study cohort

Retrospective pathology electronic database analysis was used to identify bladder biopsies/transurethral resections of the bladder (referred to here on as “biopsies”) from 2014 to 2018 performed at The Ohio State University Medical Center. Cases that had been assessed with AMACR (Dako, Santa Clara, CA, clone 13H4, dilution 1:300) and CK20 (Dako, Ks20.8, 1:200) by IHC prior to diagnosis were identified. Of these, cases with a final reported diagnosis of urothelial CIS or non-neoplastic urothelium were selected. Cases that had not been assessed with any IHC stains prior to diagnosis with a final reported diagnosis of urothelial CIS were also identified. Archived slides from all cases with AMACR performed were obtained from the surgical pathology files and were reviewed to confirm the diagnosis (by D.L.Z.). For the group with IHC performed for this study, consecutive cases with sufficient remaining tissue were selected.

The cases above comprised the following groups: 1. urothelial CIS with IHC (*n* = 22), 2. non-neoplastic urothelium with IHC (diagnosed as reactive urothelium) (*n* = 30), and 3. urothelial CIS without IHC (*n* = 20). The groups urothelial CIS with IHC and non-neoplastic urothelium with IHC represent cases in which it was difficult to distinguish urothelial CIS from non-neoplastic tissue using the H&E slides alone.

### Immunohistochemistry

Cases in the group urothelial CIS without IHC were assessed via AMACR IHC (same clone and conditions as used in clinical practice, see above) for this study. Sections (4 μm) from one representative block from each case were deparaffinized online on the Bond III instrument using ready to use dewax and 100% ethanol. Sections were then retrieved online using high pH (ER2) for 20 min. The slides were then incubated with a primary monoclonal antibody specific for AMACR with a dilution of 1:300 for 15 min. Detection was performed using the Leica Bond Polymer Refine Detection system. Prostatic adenocarcinoma was used as the positive control.

Each case, either previously stained (AMACR, CK20) or in which staining was performed for this study, was analyzed for expression (D.L.Z). Immunoreactivity was semiquantitatively evaluated as negative (luminal expression in ≤1/3 of the urothelial thickness, inclusive of umbrella cell staining), partial positive (> 1/3 but ≤2/3), or positive (> 2/3 to transurothelial). Overall intensity per case was semiquantitatively graded from 1 to 3 (1, weak; 2, moderate; 3, strong) and a mean intensity was calculated for cases which had partial positive or positive expression.

## Results

### Cohort

Diagnostic and treatment history of the cohort are provided in Table 1. Cohort characteristics of the three groups (urothelial CIS with IHC, non-neoplastic urothelium with IHC, and urothelial CIS without IHC) were similar. There was no significant difference in rate of positive cases in any group based on history of invasion, history of BCG therapy, or future invasive disease. There was no significant difference in the intensity of staining based on patient treatment or diagnostic history.

**Table 1:** Diagnostic and treatment history of the cohort

	History of urothelial CIS	History of invasive disease	History of intravesical BCG	Future urothelial CIS
Urothelial CIS with IHC (*n* = 22)	10/22 (45%)	10/22 (45%)	12/22 (55%)	5/22 (23%)
Non-neoplastic urothelium with IHC (*n* = 30)	11/30 (37%)	17/30 (57%)	15/30 (50%)	4/30 (13%)
Urothelial CIS without IHC (*n* = 20)	6/20 (30%)	8/20 (40%)	7/20 (35%)	10/20 (50%)

*BCG*, Bacillus Calmette-Guerin; *CIS*, Carcinoma in situ; *IHC*, Immunohistochemistry

### Urothelial CIS with IHC

**Fig. 1:**
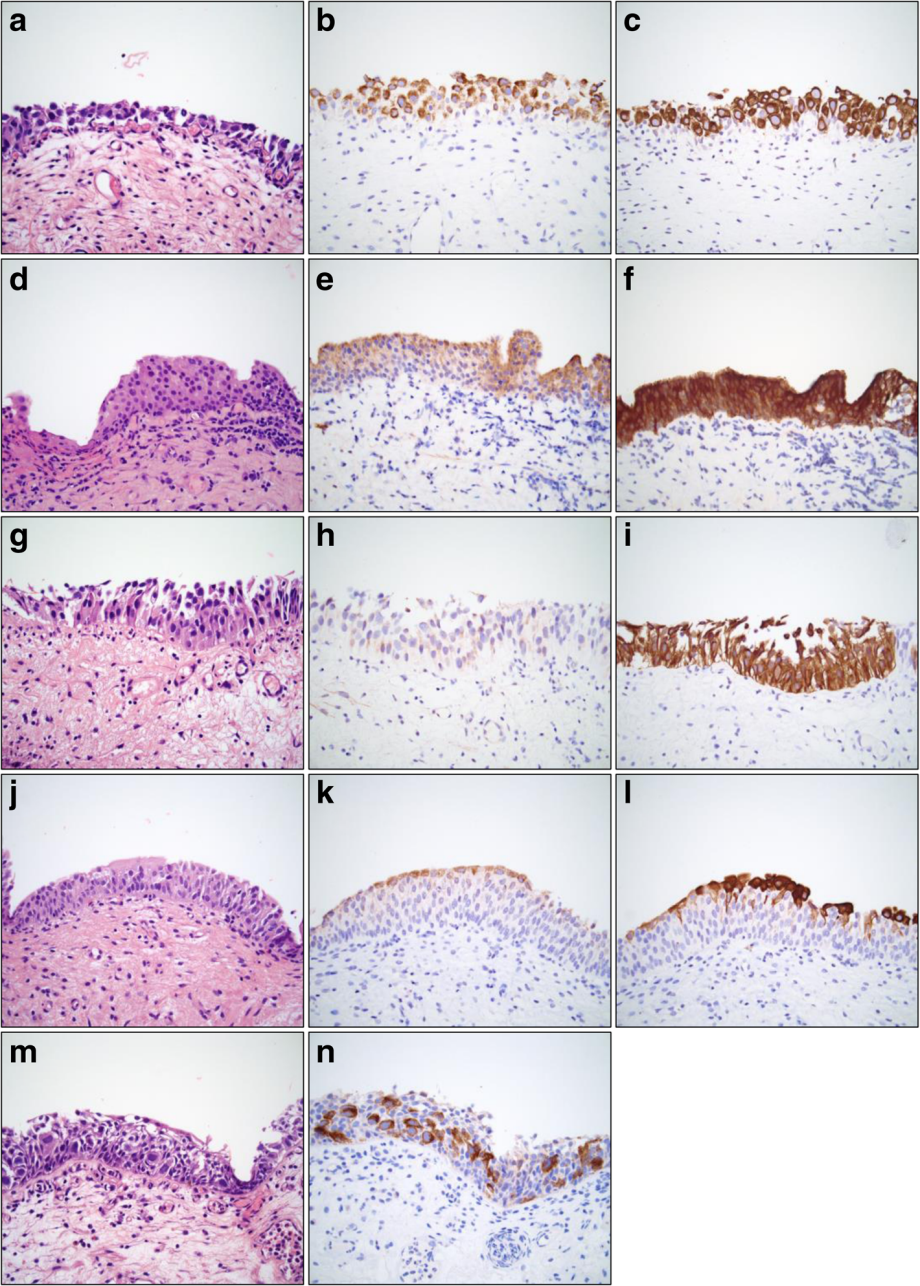
Photomicrographs of urothelial carcinoma in situ (CIS) and non-neoplastic urothelium (all images × 400 magnification). **a-c**, CIS that was equivocal on H&E (**a**) showing strong, transurothelial expression with AMACR (**b**) and CK20 (**c**). **d-f**, CIS equivocal on H&E (**d**) with transurothelial, moderate intensity staining with AMACR (**e**) and transurothelial, strong staining with CK20 (**f**). **g-i**, CIS equivocal on H&E (**g**) with minimal AMACR expression (**h**) yet transurothelial, strong CK20 expression (**i**). **j-l**, Non-neoplastic urothelium on H&E (**j**) with surface AMACR expression (**k**) and surface CK20 expression (**l**). **m-n**, Pagetoid urothelial CIS on H&E (**m**) showing staining of malignant cells with AMACR within a background of non-neoplastic urothelium that is negative for AMACR (**n**)

Almost all cases of urothelial CIS with IHC were positive for AMACR (positive 16/22, 73%; negative 6/22, 27%) with a mean intensity of 2.4 (Fig. 1a-i). Both groups of urothelial CIS included cases of pagetoid growth pattern (1 case of urothelial CIS with IHC, 2 cases of urothelial CIS without IHC), which clearly highlighted the AMACR staining pattern (Fig. 1m-n).

Almost all cases of urothelial CIS with IHC were positive for CK20 (positive 21/22, 95%; negative 1/22, 5%), which was significantly greater than AMACR (95% vs 73%, *p* = 0.02). All cases in this group that were positive for CK20 demonstrated strong staining, with a mean intensity significantly greater than that of AMACR (3.0 vs 2.4, *p* = 0.01) (Table 2) (Fig. 1a-i). All cases positive for AMACR were also positive for CK20. Only one case of urothelial CIS with IHC did not express CK20, which was in concordance with AMACR. The patient had a history of low-grade noninvasive urothelial carcinoma and urothelial CIS, status post BCG therapy complicated by recurrent urinary tract infections. Morphology of the abnormal urothelium on additional sections showed marked nucleomegaly, loss of polarity and prominent mitotic figures. Additionally, the focus had strong and diffuse p53 reactivity, unlike the adjacent urothelium. Therefore, despite the CK20 and AMACR results, a diagnosis of CIS was rendered.

**Table 2:** AMACR and CK20 expression in urothelial carcinoma in situ and non-neoplastic urothelium

		Positive	Partial positive	Negative	Mean intensity
CIS with IHC (*n* = 22)	AMACR	16/22 (73%)	0/22 (0%)	6/22 (27%)	2.4
	CK20	21/22 (95%)	0/22 (0%)	1/22 (5%)	3.0
Non-neoplastic with IHC (*n* = 30)	AMACR	0/30 (0%)	1/30 (3%)	29/30 (97%)	2.0
	CK20	5/30 (17%)	1/30 (3%)	24/30 (80%)	2.7
CIS without IHC (*n* = 20)	AMACR	20/20 (100%)	0/20 (0%)	0/20 (0%)	2.1

*CIS*, Carcinoma in situ; *IHC*, Immunohistochemistry

Comparing CIS that was CK20 positive/AMACR positive to CK20 positive/AMACR negative yielded no significant difference in history of BCG therapy, prior CIS diagnosis or history of urothelial lesions ≥pT1. Minimal follow up precluded analysis of future diagnoses between these groups.

Similar to AMACR, pagetoid morphology was readily observable with CK20 expression.

### Non-neoplastic urothelium with IHC

No cases of non-neoplastic urothelium with IHC (diagnosed as reactive urothelium) were positive for AMACR, but 1 case showed weak partial expression of > 1/3 but ≤2/3 of the urothelial thickness (partial positive 1/30, 3%; negative 29/30, 97%) (Table 1), and expression was strongest in umbrella cells with weaker staining in the upper 1/3 of the urothelium (Fig. 1k). The single partially positive case in this group had previous diagnoses that included recurrent noninvasive papillary urothelial carcinoma (low-grade and high-grade) and urothelial CIS, status post induction BCG therapy and three rounds of maintenance BCG therapy. After the biopsy was performed, the patient received one more round of maintenance BCG therapy, and had no recurrence of disease at 24 months. Many cases (14/29, 45%) that were classified as negative (luminal expression in ≤1/3 of the urothelial thickness) did show weak surface positivity (Fig. 1j-k).

The majority of non-neoplastic cases with IHC did not express CK20 (positive 5/30, 17%; partial positive 1/30, 3%; negative 24/30, 80%). Mean intensity of CK20 in this group was 2.7 (Table 2). However, significantly more non-neoplastic cases showed positive or partial positive CK20 expression than AMACR (20% vs 3%, *p* = 0.02). The only case in this group that displayed positive AMACR expression, described above, was also positive for CK20. Like AMACR, many cases that were classified as negative did show weak surface positivity (15/24, 63%) (Fig. 1j-l).

### Urothelial CIS without IHC

All cases of urothelial CIS without IHC were positive for AMACR (20/20, 100%) with a mean intensity of 2.1. The percentage of cases with positive or partially positive AMACR expression was significantly greater for urothelial CIS without IHC compared to urothelial CIS with IHC (100% vs 73%, *p* = 0.02). The mean intensity was greater in the group urothelial CIS with IHC compared to without IHC, but the difference was not significant (2.4 vs 2.1, *p* = 0.26). The rate of positivity of AMACR was significantly lower for non-neoplastic urothelium with IHC compared to urothelial CIS without IHC (3% vs 100%, *p* < 0.01) and urothelial CIS with IHC (3% vs 73%, *p* < 0.01).

## Discussion

AMACR overexpression has been described in urothelial carcinoma in situ [19, 20]. CK20 also has increased expression in urothelial CIS [7–14]. Thus, both AMACR and CK20 may be helpful in diagnosing challenging cases of CIS. To the best of our knowledge, there are no published studies that have examined the expression of AMACR in difficult cases as a part of clinical practice and compare these results to CK20.

In straightforward cases of urothelial CIS in which the diagnosis was unequivocal on H&E light microscopy alone, we found AMACR positivity, defined as expression in > 1/3 of the apical urothelial thickness, to have 100% sensitivity. Staining was granular, cytoplasmic, and variable in strength. The sensitivity in our study was comparable to that reported by Fellegara et al. (96%) [20]. This group defined AMACR positivity by diffuse, microgranular, cytoplasmic staining confined to the superficial 2/3rd’s of the urothelium. Aron et al. reported a lower sensitivity of AMACR for urothelial CIS, with AMACR positivity in 78% of untreated urothelial CIS and 50% of post-treatment cases [19]. Aron et al. defined positive as > 5% staining, which differed from our scoring criteria and that of Fellegara et al. We found that in cases that were equivocal on H&E and required IHC to assist in the diagnosis of urothelial CIS in clinical practice, AMACR had a sensitivity of 73% (*n* = 22), significantly less than that of CIS not requiring IHC for diagnosis. Additionally, AMACR can assist in recognition of CIS with pagetoid growth, in which neoplastic cells exist among non-neoplastic cells. This particular advantage of AMACR was also described previously by Fellegara et al. [20], who found AMACR to be useful in recognizing this urothelial CIS growth pattern.

The specificity of AMACR in our investigation for non-neoplastic urothelium (diagnosed as reactive urothelium) with IHC was 97%. Only 1 case of non-neoplastic, reactive urothelium had partial positive expression of regenerating cells in a patient status post multiple rounds of BCG therapy. This was similar to the specificity reported by Aron et al. (100%) and Fellegara et al. (93%) [19, 20]. However, one potential pitfall of AMACR is that it frequently is expressed in umbrella cells or surface urothelium. Almost half (45%) of non-neoplastic cases exhibited some surface expression confined to the upper 1/3rd urothelium or umbrella cells. Previous reports described similar staining of surface epithelium in non-neoplastic cases [19, 20].

CK20 had a similar pattern of expression as AMACR but was more sensitive (95% vs 73%) and less specific (80% vs 97%). The intensity of CK20 expression was consistently strong, allowing for easier interpretation compared to AMACR, which was highly variable in intensity. In addition, CK20 shares the same advantage as AMACR in that it easily demonstrated pagetoid growth, but also shared the potential pitfall of often showing surface expression in non-neoplastic urothelium. The only published study that directly compared the expression of CK20 with AMACR in urothelial CIS found CK20 to be less sensitive than AMACR (79% vs 96%) but more specific (100% vs 93%) [20]. However, there was no comparison of AMACR to CK20 in difficult cases in which IHC was performed in clinical practice prior to diagnosis, as was done in our study. Based on our results, CK20 was superior to AMACR for the indication of identifying CIS in challenging cases due to its higher sensitivity and stronger staining intensity.

In cases of classic urothelial CIS, AMACR sensitivity was 100%, and thus there was no difference in rate of positive staining based on BCG therapy history, and although cases with a history of BCG therapy did have a higher mean intensity, the difference was not significant. Fellegara et al. also did not observe a difference in AMACR expression based on treatment history [20]. However, this differed from the findings of Aron et al., in which pre-treatment samples had a sensitivity of 78%, while post-treatment sensitivity was 50% [19]. In our cohort, treatment was limited to BCG while in the prior two studies treatment was not specified for all cases.

## Conclusions

AMACR can be used to confirm the diagnosis of urothelial CIS with high sensitivity and specificity, both in classic urothelial CIS and in challenging cases. However, it should be noted that the sensitivity of AMACR is decreased in CIS that is equivocal on H&E. Furthermore, despite similar patterns of staining, CK20 showed stronger and more consistent intensity of staining, making it easier to interpret than AMACR. Therefore, CK20 is recommended instead of AMACR for this indication.

## Data Availability

Data and materials of this work are available from the corresponding author on reasonable request.

## Abbreviations

AMACR: Alpha-methylacyl-CoA-racemase
BCG: Bacillus Calmette-Guerin
CIS: Carcinoma in situ
H&E: Hematoxylin and eosin
IHC: Immunohistochemistry
